# Supplementary material for: RNA Editing Alters miRNA Function in Chronic Lymphocytic Leukemia
Source: Cancers (Basel). 2020 May 5;12(5):1159. doi: 10.3390/cancers12051159 (PMC7280959; doi:10.3390/cancers12051159)
Supplement: Supplementary file 1 [file cancers-12-01159-s001.zip › cancers-771118-after proof-supl.pdf]

A

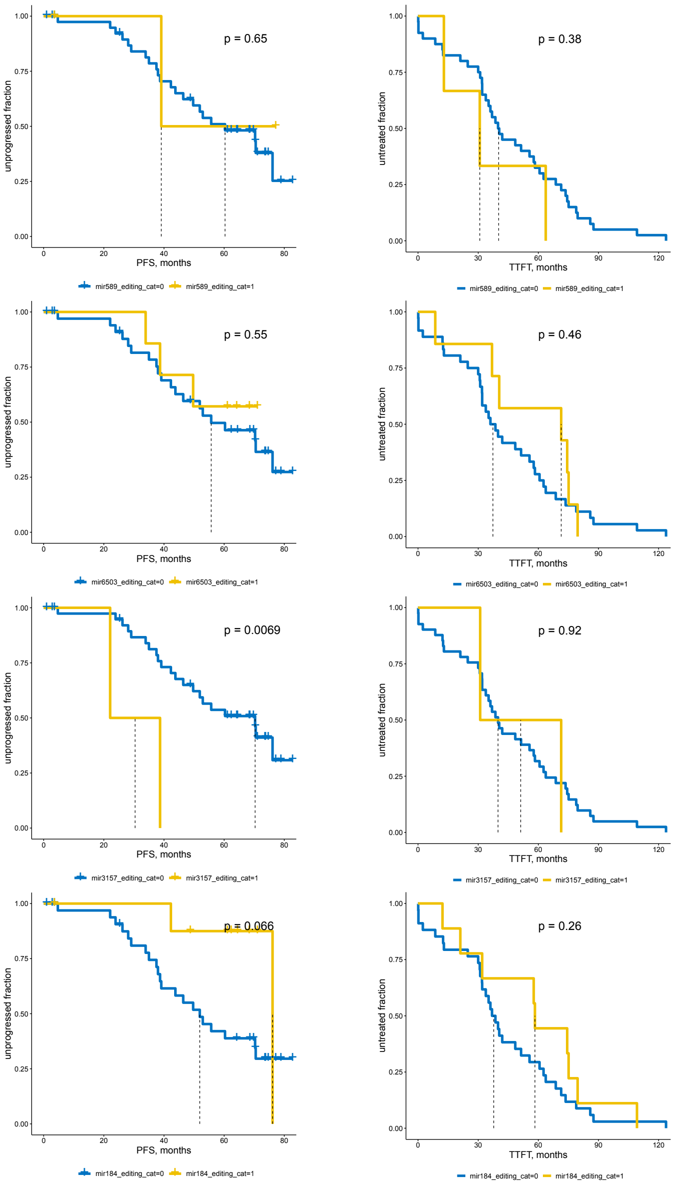

B

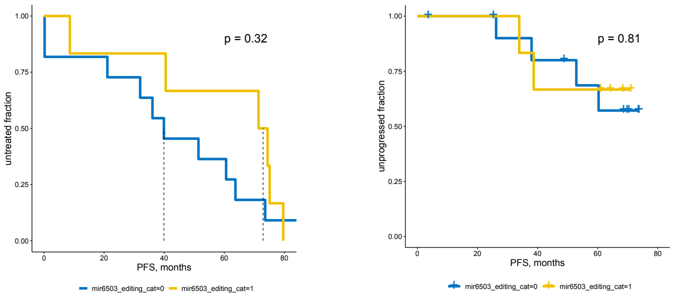

Figure S1. Kaplan-Meier plots for progression free survival (PFS) and time to first treatment (TTT) for individual editing events in CLL
